# Comparison of Autografts and Biodegradable 3D-Printed Composite Scaffolds with Osteoconductive Properties for Tissue Regeneration in Bone Tuberculosis

**DOI:** 10.3390/biomedicines11082229

**Published:** 2023-08-08

**Authors:** Tatiana I. Vinogradova, Mikhail S. Serdobintsev, Evgenia G. Korzhikova-Vlakh, Viktor A. Korzhikov-Vlakh, Alexander S. Kaftyrev, Natalya M. Blum, Natalya Yu. Semenova, Dilyara S. Esmedlyaeva, Marina E. Dyakova, Yulia A. Nashchekina, Marine Z. Dogonadze, Natalia V. Zabolotnykh, Petr K. Yablonsky

**Affiliations:** 1 Saint-Petersburg State Research Institute of Phthisiopulmonology, Ministry of Health of the Russian Federation, Ligovskiy pr. 2–4, St. Petersburg 191036, Russia; vinogradova@spbniif.ru (T.I.V.); osteolog@mail.ru (M.S.S.); niif5@yandex.ru (A.S.K.); diljara-e@yandex.ru (D.S.E.); marinadyakova@yandex.ru (M.E.D.); marine-md@mail.ru (M.Z.D.); zabol-natal@yandex.ru (N.V.Z.); glhirurg2@mail.ru (P.K.Y.); 2 Institute of Macromolecular Compounds, Russian Academy of Sciences, Bolshoy pr. 31, St. Petersburg 199004, Russia; vlakh@hq.macro.ru; 3 Institute of Chemistry, Saint-Petersburg State University, Universitetskiy pr. 26, St. Petersburg 199034, Russia; 4 Department of Pathological Anatomy, S.M. Kirov Military Medical Academy, Botkinskaya str. 21/2, St. Petersburg 194044, Russia; blumn@mail.ru; 5 Interregional Medical Center, Oleko Dundich str. 8/2, St. Petersburg 192283, Russia; natyciel87@gmail.com; 6 Institute of Cytology, Russian Academy of Sciences, Tikhorezkii pr. 4, St. Petersburg 194064, Russia; ulychka@mail.ru

**Keywords:** biocomposite scaffolds, biomaterials, bone tuberculosis, bone regeneration

## Abstract

Tuberculosis remains one of the major health problems worldwide. Besides the lungs, tuberculosis affects other organs, including bones and joints. In the case of bone tuberculosis, current treatment protocols include necrectomy in combination with conventional anti-tuberculosis therapy, followed by reconstruction of the resulting bone defects. In this study, we compared autografting and implantation with a biodegradable composite scaffold for bone-defect regeneration in a tuberculosis rabbit model. Porous three-dimensional composite materials were prepared by 3D printing and consisted of poly(ε-caprolactone) filled with nanocrystalline cellulose modified with poly(glutamic acid). In addition, rabbit mesenchymal stem cells were adhered to the surface of the composite scaffolds. The developed tuberculosis model was verified by immunological subcutaneous test, real-time polymerase chain reaction, biochemical markers and histomorphological study. Infected animals were randomly divided into three groups, representing the infection control and two experimental groups subjected to necrectomy, anti-tuberculosis treatment, and plastic surgery using autografts or 3D-composite scaffolds. The lifetime observation of the experimental animals and analysis of various biochemical markers at different time periods allowed the comparison of the state of the animals between the groups. Micro-computed tomography and histomorphological analysis enabled the evaluation of osteogenesis, inflammation and cellular changes between the groups, respectively.

## 1. Introduction

Tuberculosis (TB) is still one of the major health problems [1,2]. According to the World Health Organization (WHO), 10.6 million people fell ill with tuberculosis and 1.6 million died from this disease worldwide in 2021 [3]. Mortality from tuberculosis took 2nd place after COVID-19 in the list of infectious diseases and 13th place in the list of the leading causes of death.

Tuberculosis is caused by *Mycobacterium tuberculosis (M. tuberculosis)*, which mainly localizes in the lungs (pulmonary TB), but other parts of the body can also be affected (extrapulmonary TB) [2]. As a result of the dissemination of infection from the lungs through the blood and lymphatic systems, tuberculosis may affect the lymph nodes, pleural cavity, genitourinary tract, abdominal organs, and central nervous system structures, as well as bones and joints [4,5,6]. In addition, cases of tuberculosis affecting the eyes and middle ear are also known [7,8]. Bone tuberculosis, also called tuberculous osteitis (osteomyelitis), accounts for 1 to 3% of all tuberculosis cases [9,10,11].

Traditional treatment for tuberculous osteitis is based on surgical removal of necrotic bone tissue (necrectomy) combined with anti-TB chemotherapy [10]. The latter often causes adverse side effects [12]; to minimize this, the use of drug delivery systems is widely considered [13,14,15]. As an additional effective approach to treat multidrug-resistant tuberculosis, the application of mesenchymal stem cells (MSCs) can also be a matter of choice. Given the immunomodulatory and tissue-preserving abilities of MSCs, their use can help in the clinical treatment of patients by transforming chronic inflammation into a productive immune response [16,17]. Numerous animal and human studies indicate an increasing role of MSCs in the regulation of cell-mediated immunity in tuberculosis [18]. Furthermore, a number of studies have shown the osteogenic properties of MSCs in the formation of new bone after surgical removal of bone tissue affected by tuberculosis in an animal model [19,20].

Today, the high efficiency of therapy is aimed at the comprehensive treatment of tuberculosis, including not only surgical removal and perioperative anti-TB treatment, but also the reconstruction and repair of the resulting bone defects [21,22]. In this regard, an important role in reconstructive surgery in bone tuberculosis is assigned to the development of new osteosubstituting materials capable of replacing the conventionally used autologous bone material. Furthermore, the search for new factors capable of specifically altering the natural regenerative processes in damaged bone tissue is essential for improving bone repair process.

In recent years, a number of studies have reported the development and investigation of various materials for application as bone substitutes [23,24,25,26]. Modern approaches include the development of biocompatible scaffolds with mechanical characteristics close to bone and capable of supporting the biomineralization and growth of the new vessels. To date, a wide range of scaffolds based on bioceramics [27,28], porous polymeric [23,26] and composite materials [29,30,31,32], have been developed. Among the reported polymer scaffolds, 3D-printed materials [33] based on biocompatible and biodegradable thermoplastic aliphatic polyesters, such as poly(lactic acid) (PLA), poly(lactide-co-glycolide) (PLGA), poly(ε-caprolactone) (PCL), poly(3-hydoxybutyric acid) (PHB), etc., have demonstrated the appropriate mechanical properties. The latter can be additionally enhanced by the use of various nanoparticles as fillers, such as hydroxyapatite [34,35], calcium carbonate [36], graphene oxide [37], montmorillonite [38], nanocrystalline cellulose [39], and others. The biological properties of scaffolds are modulated by the introduction of various polymeric (polypeptides, glycolpolymers, etc.) [40,41] and biological (MSCs, bone morphogenetic proteins, transforming growth factors beta, RGD-peptides, etc.) components [42,43,44,45] to enhance cell adhesion, proliferation and osteodifferentiation as well as biomineralization and bone tissue formation.

In the case of bone tuberculosis, several studies have focused on developing scaffolds for complex therapy. In particular, Wardhania et al. developed nanohydroxyapatite/streptomycin/gelatin-based injectable composite as a bone substitution in the case of spinal tuberculosis [46]. Huang et al. reported the fabrication of poly(lactide-co-glycolide)-based (PLGA) scaffolds containing conjugated anti-tuberculous drug isoniazid and tricalcium phosphate for sustained local treatment and tissue regeneration in bone tuberculosis complex therapy [47]. Min at al. manufactured the 3D-printed macro/meso-porous bioceramic composite scaffolds loaded with two anti-tuberculous drugs, e.g., isoniazid and rifampin [48]. The resulting scaffolds showed prolonged drug release both in vitro and in vivo. In the latter case, the drug concentration in the defect area was maintained above the minimum inhibitory drug concentrations up to 12 weeks after surgery, while the drug concentration in the blood was extremely low. A scaffold consisting of nanohydroxyapatite/polyamide 66 composite with immobilized isoniazid was recently reported by Xie et al. [49]. The experiment on bone-defect repair in rabbits showed that a composite scaffold provided both antituberculosis and bone-regeneration activity.

Recently, we developed 3D-printed composite scaffolds based on PCL filled with nanocrystalline cellulose (NCC) modified with poly(glutamic acid) (PGlu) and investigated them as implants for bone regeneration in a femur bone of healthy rabbits [43]. The combination of the developed osteoconductive composites with MSCs provided the best osteoregeneration effect compared to the control scaffolds.

In this work, we investigated PCL/PGlu-NCC scaffolds with adhered rabbit bone MSCs (PCL/PGlu-NCC + rMSCs) to repair defects formed after the necrectomy of bone affected by tuberculosis. The results obtained in the experiment on implantation with 3D-printed composite scaffolds was compared with those on autograft surgery in a rabbit femur bone tuberculosis model. Lifetime observation of the animals and analysis of various biochemical markers at different time periods allowed the comparison of the state of the animals between the groups. Micro-computed tomography and histomorphological analysis enabled the evaluation of osteogenesis, inflammation and cellular changes between the groups, respectively.

## 2. Materials and Methods

### 2.1. Materials

NCC was purchased from Blue Goose Biorefineries Inc. (Saskatoon, SK, Canada). PCL (M_w_ = 155,000; PDI = 1.53; η = 1.3 dL/g) was synthesized by the ring-opening polymerization (ROP) of ε-caprolactone as described elsewhere [50]. PGlu (*M_w_* = 2100, *Đ* = 1.05) containing 22% (according to NMR) of the residual benzyl groups was synthesized by ROP of N-carboxyanhydride of γ-benzyl ester of glutamic acid according to a previously published protocol [51]. NCC was modified with PGlu according to a previously developed protocol [51].

*M. tuberculosis H37Rv* strain was received from the collection of the Russian Ministry of Health Research Center for the Examination of Medical Devices. Rabbit bone marrow MSCs were isolated and cultivated as described elsewhere [43]. For rMSC cultivation DMEM (Gibco, Billings, MT, USA) supplemented with 10% FBS (HyClone, Logan, UT, USA), penicillin (50 U/mL) and streptomycin (50 mg/mL) (Biochrom, Berlin, Germany) were used as growth medium.

Chinchilla rabbits were purchased from Rappolovo Laboratory Animal Breeding Station of the National Center Kurchatov Institute and kept in a certified vivarium at the St. Petersburg Research Institute of Phthisiopulmonology of the Ministry of Health of Russia in accordance with the European Convention for the Protection of Vertebrate Animals used for experimental and other scientific purposes (Strasburg, 1986).

### 2.2. Preparation of 3D-Printed Composite Scaffolds with Adhered rMSCs

CAD-modeled scaffolds were printed from the PCL/PGlu-NCC blend containing 10 wt% of the filler (PGlu-NCC) using the GeSim Bioscaffolder 3D printer (Radeberg, Germany) equipped with pneumatic extruder. The blend was pre-melted before 3D-printing for 20 min at 80 °C. The conditions used for 3D-printing were set as follows: cartridge temperature 80 °C; bed temperature 35 °C; diameter of the printing metal nozzle 0.4 mm; printing pressure 500 kPa at a printing speed of 0.1 mm/s. The scaffolds had a cylindric shape and were 4 mm in diameter, and 3 mm in height.

Before biological experiments, 3D-printed composite scaffolds were sterilized with 70% ethanol solution for 3 h and then washed several times with sterile PBS. To adhere rMSCs on the surface of the scaffolds, 2 × 10^5^ cells were seeded on each scaffold placed in a sterile 24-well plate. Scaffolds were incubated for 2 h, and then 1 mL of growth medium was added and cells were cultivated for 4 days in CO_2_-incubator (5% CO_2_) at 37 °C.

### 2.3. Experiments In Vivo

The study included the results of dynamic observation of 31 chinchilla rabbits. The present study was authorized by the Independent Ethical Committee of the St. Petersburg Research Institute of Phthisiopulmonology of the Ministry of Health of Russia.

During the acclimatization period (10 days), daily clinical examination of the animals was performed. The criteria for inclusion of the animals in the experiment: positive dynamics of body weight during the quarantine period, absence of visible symptoms of the disease.

The rabbits were kept one at a time in stainless steel cages type NYA K (S = 4200 cm^2^), equipped with feeders and standard drinkers under controlled environmental conditions. Light regime: 12 h light, 12 h dark. Temperature was kept between +23 and +25 °C, and relative humidity 50–70%. Air exchange was maintained by means of combined extract and input ventilation; air sterilization was performed daily using quartz treatment. Food and water were given ad libitum.

The animals were randomized using an online random number generator into groups according to the variant of treatment. The study included three experimental groups:

- Group 1: infection control (IC)—infection, no surgical operation, no treatment, (*n* = 4);
- Group 2: autoplastic (AP)—infection, necrectomy, autoplastic surgery, anti-TB treatment, (*n* = 12);
- Group 3: scaffold implantation (SI)—infection, necrectomy, plastic surgery with the composite scaffold, anti-TB treatment, (*n* = 15).

#### 2.3.1. Tuberculosis Modeling

Tuberculosis ostitis was modeled in the distal metaepiphysis of the femur according to the earlier developed method based on inoculating a standardized culture of *M. tuberculosis H37Rv* [52]. Briefly, the method involved several steps: (1) general anesthesia, (2) soft-tissue dissection and the formation of a defect in the bone, (3) inoculation of *M. tuberculosis* with a predetermined concentration, (4) filling with a cement seal, and suturing of the wound.

Anesthetic treatment included anesthesia with Zoletil (zolozepam + tiletamine, Virbak SA, Carros, France) in a dose of 25 mg/kg intravenously into the marginal auricular vein and myorelaxant Rometar (Xylazinum, Bioveta, Ivanovice na Hane, Czech Republic) as a 2% solution intramuscularly in 1.0–1.5 mL.

The position of the rabbit was on its back. The limbs of the rabbit were fixed in the maximum extended position. Fur on the skin in the area of the knee joint was removed and skin was treated with 2% alcohol iodine solution. The skin and soft tissues were dissected at the anterior edge of the collateral ligament up to the bone and moved downwards and medially with the raspator together with the periosteum. A canal of 4 mm in diameter and 5 mm in length was drilled in the distal metaepiphysis of the femur bone. Bleeding was stopped by tamponade of the canal with 3% hydrogen peroxide solution.

*M. tuberculosis* suspension of a given concentration (10^7^ CFU) was injected into a hemostatic sponge, which was placed into the formed bone canal. The external surface of the bone defect was isolated from the paraosseous soft tissues with a cement seal (Synicem 1 purchased from Synimed, Chamberet, France), and the wound was sutured in a layer-by-layer manner tightly.

Within 5 days after infection, antibiotic treatment with Cefazolin (Pharmasintez, Ussuriysk, Russia) in a dose of 50,000 units/kg, 1.5 mL intramuscularly was performed.

#### 2.3.2. Tuberculosis Verification

18 days after modeling the foci of specific inflammation in animals, their development was verified by delayed-type hypersensitivity reaction with commercially available recombinant tuberculosis allergen Diaskintest (Generium, Moscow, Russia). A volume of 0.1 mL of Diaskintest in physiological solution at a concentration of 2 µg/mL was injected intradermally into the shaved area on the back.

In addition, determination of the DNA fragments of *M. tuberculosis* in the rabbit bone tissue samples, obtained during the necrectomy of the destructive foci in groups 2 and 3, was performed by real-time polymerase chain reaction (RT-PCR) using Bio-Rad CFX96 Real-Time System (Bio-Rad Laboratories, Hercules, CA, USA), and a standard Amplitub-RV kit for tuberculosis analysis (Syntol, Moscow, Russia), according to the manufacturer protocol.

#### 2.3.3. Treatment and Plastic Surgery

After infection, animals from groups 2 and 3 received isoniazid (10 mg/kg, MosChemPharmPreparates, Moskow, Russia), pyrazinamide (15 mg/kg, Pharmsintez, Moscow, Russia) and ethambutol (20 mg/kg, Shreya Life Sciences, Mumbai, India). After three weeks of treatment, necrectomy of the area of specific inflammation followed by the autoplastic surgery or implantation with composite scaffolds of the formed defect was performed in groups 2 and 3, respectively. In the case of autografting, a cortical bone graft of 4 mm in length and 3 mm in diameter was harvested from the femur proximal to the area of infection. The area of autograft extraction was filled with a cement seal, while the post-surgery bone defect was replaced with autograft. In the case of scaffold implantation, the bone area after necrectomy was filled with a composite scaffold with adhered rMSCs. The wound was sutured layer-by-layer tightly. Further antituberculosis therapy was continued until the end of the experiment.

### 2.4. Biochemical Analysis

The severity of the infection was assessed by biochemical indices of the inflammatory response and markers of bone tissue metabolism (baseline, 18 days after infection; 2, 4, and 6 months after the start of treatment). The following parameters were determined in serum: activity of purine metabolism enzymes, namely, the general adenosine deaminase (ADA) and its isoenzymes (ADA-1 and ADA-2) [53]; the level of acute phase reactants (RPE)—ceruloplasmin (CP) concentration (Ravin method); albumin (AL) concentration, total protein (TP)—were determined by biochemical analyzer SynchronCX5 PRO (BeckmanCoulter, Oklahoma City, OK, USA) using BeckmanCoulter reagents and the standard protocol of the manufacturer; and the activity of the destruction marker elastase (EL) [54], non-specific alkaline phosphatase (ALPL) and the receptor activator of nuclear factor kappa-B ligand (RANKL) were determined by ELISA according to the protocol of the manufacturer (Cloud-Clone Corp, Wuhan, Hubei, Chia; and BT Lab, Shanghai, China).

### 2.5. Microcomputed Tomography

Before histomorphological analysis, bone specimens fixed in 10% solution of neutral buffered formalin (pH 7.4) were analyzed via microcomputed tomography (micro-CT) using a Bruker MicroCT SkyScan 1172 instrument (Billerica, MA, USA). A RadiAnt DICOM Viewer (version 20.2, Medixant Maciej Frankiewicz, Poznań, Poland), MicroDicom (MicroDicom, Sofia, Bulgaria), SkyScan DataViewer-v.1.5.2.4 and ctVOX-v.3.2.0r1294 (Brucker micro-CT, Billerica, MA, USA) software were used for the evaluation of the micro-CT images (4096 × 4096 pixels). The presence/absence of the bone trabeculae and structural bone beams, and a bone resorption zone at the point of contact with the implant, as well as the formation/absence of cortical closure plate and marginal bone growths in the area of the cortical closure plate of the bone at the site of its contact with the implant were analyzed.

For the analysis of defect size (mm^2^) dynamics, a set of 16 micro-CT bone slice images at the defect site were used. The defect area was assessed by an ellipse measure tool with application of MicroDicom viewer. Bone repair at each time point was calculated according to the following equation:

(1) $$Bone\;repair\;\left(\%\right)=\frac{\left(Defect\;size\;of\;infected\;control-Defect\;size\;at\;timepoint\right)}{Defect\;size\;of\;infected\;control}\times 100$$

### 2.6. Histomorphological Analysis

After micro-CT analysis, the distal diaphyseal–epiphyseal segments of the femur were cut off and decalcified using a TBD-2 solution (ThermoFischer, Waltham, MA, USA) for 14–21 days. When slices were possible, the bone was cut into slices no thicker than 0.4 mm and placed into histological cassettes for further decalcification until complete softening, an average of about 10–14 days. Dehydration and paraffin treatment were performed using a standardized procedure in an Excelsior AS automatic histological processor (ThermoFisher, Waltham, MA, USA) in a ready IsoPREP solution (Biovitrum, St. Petersburg, Russia) and HISTOMIX paraffin medium (Biovitrum, St. Petersburg, Russia). The 3–5 µm thick slices were made using an HM 325 rotary microtome (Thermo, Waltham, MA, USA). Then, they were subsequently deparaffinized, dehydrated, and stained according to the standardized hematoxylin–eosin and Ziehl–Neelsen methods in accordance with the manufacturer’s recommendations (Biovitrum, St. Petersburg, Russia). The results were analyzed with an optical microscope at different magnifications (×100, ×200 and ×1000) and digitalized. An examination included the evaluation of the localization of the pathological process, presence or absence of edema, granulation tissue and mesenchyme in the injury zone, active/inactive osteoblasts and the formation of the new bone beams and vessels.

The bone coverage was evaluated as based on the obtained images of histological sections at the defect site with application of the ORBIT image analyzer, Version 3.64 (developed by Manuel Stritt in Idorsia Pharmaceuticals Ltd., Allschwil, Switzerland). Ten images were taken for each sample. The calculation of bone coverage was performed according to the following formula:

(2) $$Bone\;coverage\;\left(\%\right)=\frac{Bone\;area}{Whole\;tissues\;area}\times 100$$

### 2.7. Statistical Analysis

Statistical analysis was performed with Statistica 7.0 software package (StatSoft Inc., Tulsa, OK, USA). Sample size (*n*) was calculated as based on the results of pilot biochemical tests with the application of standard Equation (1) [55]:

(3) $$n=\frac{{\left(z_{\alpha }+z_{\beta }\right)}^{2}}{\delta^{2}}\sigma^{2}$$

where $z_{\alpha }$ and $z_{\beta }$ are critical *z* values of the standard normal distribution (1.96 and 0.84, correspondingly); δ is the difference between measured values; and δ is a standard deviation obtained from preliminary experiments. A one-tailed direction of effect with effect size 0.7, significance level 0.05 and power 0.8 were assumed for sample size assessment.

The difference between the mean values of the calculated defect size area and the bone coverage for the autograft and scaffold at different time points was analyzed with the application of a two-way ANOVA using the GraphPad by Prism version 9.2.0 (San Diego, CA, USA).

The nature of sample data distribution was determined. In the case of deviation from normal distribution (by Shapiro–Wilk criterion) the median (Me), as well as first and third quartiles [Q1; Q3] were calculated. The significance of differences was assessed using a Mann–Whitney U test and Kruskal–Wallis H test (one-way ANOVA on ranks). The presence of correlations was determined by the calculation and estimation of the Spearman coefficient. Values of *p* ≤ 0.05 were considered statistically significant.

## 3. Results

### 3.1. Scaffolds

The scaffold preparation conditions and its composition optimization have been described in our previous papers [43,56]. In short, the applied composite scaffolds were obtained by an extrusion-based 3D-printing technique with the application of PCL blended with 10 wt% NCC covalently modified with PGlu (PCL/PGlu-NCC). The 3D-printed scaffolds possessed a cylindrical shape (4 mm in diameter and 3 mm in height). During printing, the filaments between the layers were overlapped in the opposite direction, forming a mesh structure. The total scaffold porosity was 61 ± 2%.

The obtained 3D-printed PCL/PGlu-NCC scaffolds were characterized with a compression modulus (*E*) equal to 101 ± 29 MPa [43]. Such mechanical properties are suitable for repairing defects of human trabecular bones. It is known that their compression modulus is close to 12 MPa [57]. According to the previous in vitro study, PCL/PGlu-NCC scaffolds supported MSCs adhesion, proliferation, osteodifferentiation and biomineralization [43]. Based on the previous positive results, the PCL/PGlu-NCC 3D-printed scaffolds used in this study were seeded with rMSCs (PCL/PGlu-NCC + rMSCs) just before surgery.

### 3.2. Tuberculosis Modeling, Verification and Treatment

Healthy Chinchilla rabbits were infected by *M. tuberculosis* and randomly divided into three groups. Group 1 was an infection control (IC), which received neither treatment nor surgery. Group 2 represented a group of animals that were infected, received antituberculosis treatment, necrectomy, and autoplastic surgery. The difference between group 3 and group 2 was that the plastic surgery was performed using a PCL/PGlu-NCC + rMSCs composite scaffold instead of an autograft.

**Figure 1:**
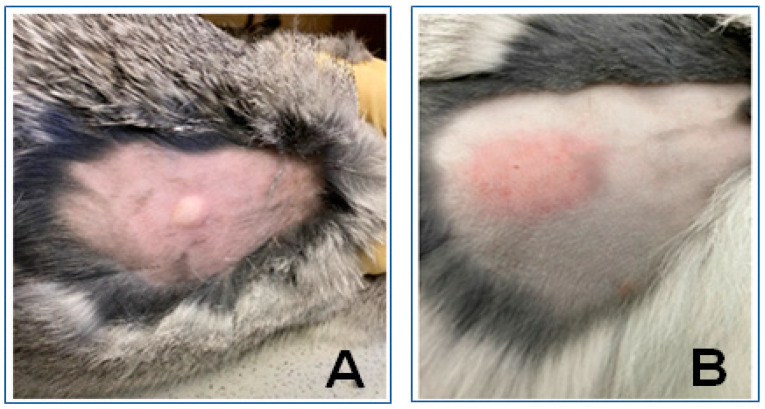
Immunological test (Diaskintest^®^) for diagnostics of tuberculosis infection (subcutaneous injection in back of animal): (**A**) subcutaneous injection on day 18 after infection; (**B**) local skin hyperemia, indicting positive result (successful infection with *M. tuberculosis*), three days after injection.

The generated tuberculosis model was verified by an immunological subcutaneous test based on the antigen injection followed with the delayed-type skin hypersensitivity reaction. For this, the commercially available test-system Diaskintest^®^ (Generium, Moscow, Russia) based on recombinant tuberculosis allergen was used. All infected animals were positive in response to Diaskintest^®^, which confirmed the successful development of the tuberculosis process (Figure 1). Moreover, *M. tuberculosis* was detected by real-time PCR in all bone tissues extracted during necrectomy from the pathological foci of rabbits.

Both autoplastic and implantation with the 3D-printed composite scaffolds were carried out under aseptic conditions after three weeks of infection. Throughout the experiment, the rabbits were monitored for weight and a number of biochemical markers (Section 3.2). In contrast to control group, in which the rabbits lose their weight, the animals in both experimental groups add their weight (Table 1). At 2, 4 and 6 months after infection, animals were euthanized and the affected untreated and implanted bones were extracted for micro-CT (Section 3.4), as well as histomorphological analysis (Section 3.5).

### 3.3. Monitoring of Biochemical Markers

By day 18 post-infection, a statistically significant decrease in RANKL concentration, accompanied by a tendency to decrease ALPL level, was observed in all groups as compared to the initial values (Table 2). At the same time, there was a decrease in the level of total protein, along with an increase in the level of CP and EL, which are an acute phase reactant and a marker of destruction activity, respectively. A positive correlation (r = 0.37; *p* = 0.05) was detected between the latter two indices. Other studied indices did not differ statistically from the initial values (Table 2).

Two months after infection, rabbits of group 1 (infected and untreated) showed a statistically significant increase in RANKL, compared with the initial values. At the same time, a high level of CP remained, along with a tendency to increase the activity of total ADA, ADA-1 and ADA-2, indicating the preservation of the activity of the inflammatory response.

In group 2 (rabbits with autologous surgery and anti-TB treatment), two months after infection, there was a statistically significant decrease in CP levels compared both with initial values and with values registered 18 days after infection. Along with this, there was a significant increase in the activity of total ADA due to ADA-1. And a negative association (r = −0.78; *p* = 0.002) was found between the activity of ADA-2 and elastase. Four months after surgery, a significant increase in the concentration of non-specific alkaline phosphatase (ALPL) was registered compared with all previous periods of observation (*p* = 0.04). Low CP concentrations persisted at this time, with CP decreasing compared to the 2-month observation period (*p* = 0.04). Along with this, there was an increase in total protein level due to albumin fraction compared to all previous periods of analysis (*p* = 0.005 and *p* = 0.009, respectively). The activity of purine metabolism enzymes (total ADA and ADA-1) remained high. Six months after infection, the concentration of bone-tissue metabolism markers remained multidirectional: a low concentration of ALPL was accompanied with a high concentration of RANKL.

In group 3 (rabbits with composite scaffold and anti-TB treatment), two months after infection, there was a statistically significant decrease in the concentration of RANKL compared to the initial values. On the contrary, the activity of EL was higher not only than the initial values, but also the values registered on the 18th day after infection. Moreover, in this case, the EL level was significantly higher than in group 1 (IC) and group 2 (AP). The level of CP was lower than on day 18 after infection and lower than in group 1 (IC). Similar to group 2 (AP), ADA activity, due to ADA-1, was also increased. Correlations (r = 0.52; *p* = 0.046; r = 0.68; *p* = 0.005, respectively) were found between the albumin level and ADA-1, elastase activity. ALPL concentration was 2.7 times lower than in the group with autoplastic (group 2). As for the enzymes of purine metabolism, only ADA-1 increased compared with the initial values. The correlations between ADA activity and albumin level (r = 0.69; *p* = 0.03) were revealed. Six months after infection, most of the studied biochemical parameters reached the initial values. The activity of ADA-2 increased statistically significantly compared with the previous periods of observation (*p* = 0.02). Although there was a tendency for the ALPL concentration to increase compared to baseline data, it was two times lower (differences were not statistically significant) than in group 2. Correlation analysis revealed the relationship between the total ADA activity and elastase (r = −0.9; *p* = 0.037) and between the activity of ADA-2 and concentration of RANKL (r = −0.9; *p* = 0.037).

### 3.4. Micro-Computed Tomography

**Figure 2:**
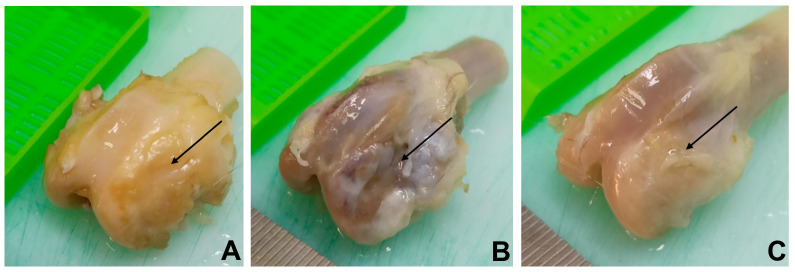
Images of rabbit femur bones with visualization of the surgical intervention sites (indicated by black arrow): (**A**) infection control (group 1); (**B**) autografting (group 2) and (**C**) implantation with PCL/PGlu-NCC + rMSCs scaffold (group 3).

**Figure 3:**
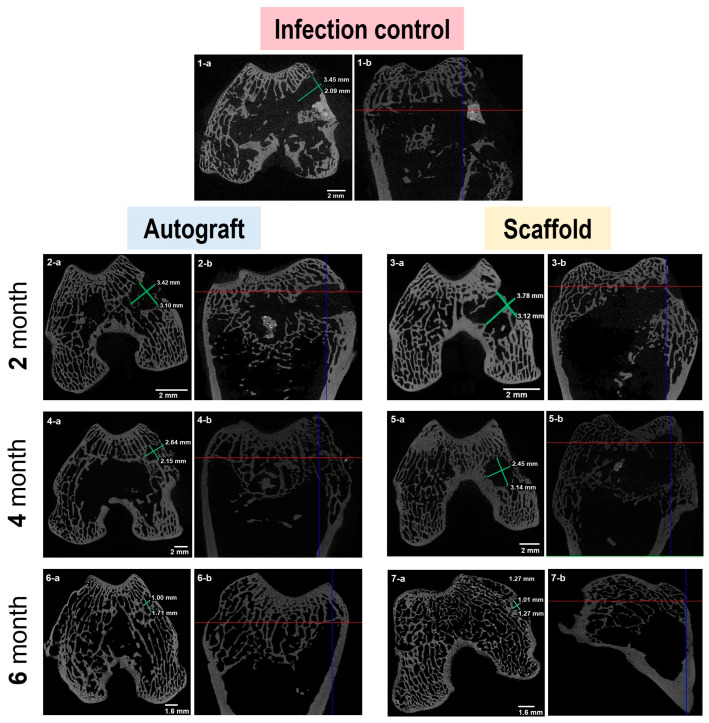
Micro-CT images for bone specimens extracted after 2, 4 and 6 months of tuberculosis infection. Axial (**1-a**–**7-a**) and corresponding sagittal (**1-b**–**7-b**) projections of bone are presented. The defect site size measurements allowed us to show bone formation process.

Typical images of bone fragments extracted from rabbits at different time periods during the experiment are shown in Figure 2. The bone regeneration process was visualized by micro-CT measurements of extracted bone specimens. One can observe (Figure 3 and Figure S1 in Supplementary Materials) the increasing amount of the bone tissue within the area of surgical intervention after 4 (Figure 3(4-a,4-b,5-a,5-b)) and 6 (Figure 3(6-a,6-b,7-a,7-b)) months of the experiment in the case of the autografting and scaffold application.

To compare osteoregeneration after different variants of treatment (infection control, autografting and scaffold implantation), micro-CT data were analyzed to assess several qualitative markers indicating changes in the bone formation and resorption processes. In particular, the presence/absence of bone trabeculae growing into the scaffold, cortical closure plate, sclerosis area along the defect, marginal bone growths and bone resorption area, as well as the structure of bone beams were assessed. The results of micro-CT data analysis are summarized in Table 3. As expected, in group 1 corresponding to the infection control, no bone formation accompanied with bone resorption was observed during both study periods. The analysis of micro-CT images of bones from group 2 (autoplastic surgery) revealed the formation of new bone structures already two months after surgery and the further development of this process over time. A similar tendency was detected for the composite scaffold implantation (group 3). The sprouting of the new bone trabeculae in the scaffold, bridging of the defect area and the formation of bone callus confirmed the biocompatible and osteogenic nature of the used composite scaffold. At the same time, unlike autoplastic surgery, no resorption zones were detected after 6 months when the composite scaffold was implanted.

### 3.5. Histomorphological Analysis

Semi-quantitative and morphometric examination was performed to clarify and visualize the evaluation in the following scope: (1) the presence and severity of specific inflammation, necrotizing zones, and cell composition of infiltration zones and the presence of *M. tuberculosis*; (2) the presence and cell composition of the formed fibrous capsule around the implant; (3) the osteogenesis, its localization and intensity (mesenchyme, active and inactive osteoblasts, newly formed bone beams) in the surgical area; and (4) cell composition of bone marrow tissues surrounding the implant area.

#### 3.5.1. Group 1: Infection Control

**Figure 4:**
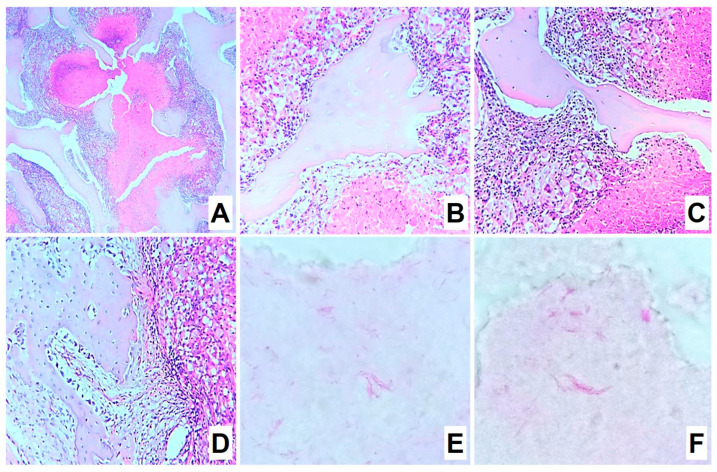
Morphological changes in group 1 after 2 months of infection: (**A**) fields of caseous necrosis with abundant detritus among bone beams, delimited by a shaft of epithelioid cells, macrophages, and lymphocytes (H&E-staining, ×100); (**B**,**C**) sequestered bone beams in the necrosis zone (H&E-staining, ×200); (**D**) newly growing bone beams in mesenchyme at the border of inflammation zones (H&E-staining, ×200); (**E**,**F**) numerous acid-resistant forms of mycobacteria in caseous masses (Ziehl–Neelsen stained mycobacteria, ×1000).

For infection control two months after infection, the structure of the metaepiphysis and epiphysis zones was partially preserved. Histomorphological analysis revealed extensive areas of destruction of bone beams with the formation of caseous necrosis zones with abundant detritus, demarcated by a shaft of epithelioid cells and macrophages (Figure 4A). On the periphery of the zones, a pronounced lymphoplasmacytic and macrophage infiltration among the masses of necrosis as well as many sequestered bone beams was detected (Figure 4B,C). At the same time, interbody spaces were completely replaced by inflammatory infiltrate and forming fibrosis. All foci of inflammation were at the same phase of progression, which indicated the synchronous character of the infectious pattern. At the same time, the newly growing bone beams in mesenchyme at the border of inflammation zones were visualized (Figure 4D). Furthermore, acid-resistant forms of bacteria morphologically similar to *M. tuberculosis* were detected in large numbers in Ziehl–Neelsen-stained sections (Figure 4E,F).

**Figure 5:**
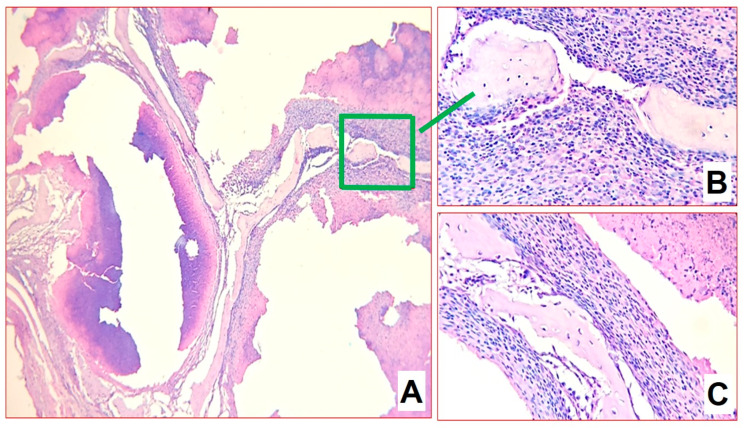
Morphological changes in group 1 after 4 months of infection (H&E-staining): (**A**) caseous-necrotic foci acquire clear outlines due to capsule formation (×100); (**B**,**C**) both pre-existing and newly formed bone beams (×200).

Four months after infection, the epiphysis architectonics were disturbed due to numerous multiple foci of caseous necrosis (Figure 5). Necrotic masses contain detritus in some places. They separated irregularly in some places by a wide layer and in some places by a narrow layer of epithelioid cells, macrophages, lymphocytes, and thin interlayers of connective tissue (Figure 5A). Capsule formation was observed; foci acquired outlines and contours. The foci were at the same stage of progression. Thinned bone beams were preserved between the foci: some of the beams were sequestered, while some had osteogenesis signs (Figure 5B,C). Bone marrow was replaced by fatty tissue. Mycobacteria were detected in small numbers.

The morphometric assessment of infiltrate cellularity allowed us to show a significant decrease in the number of specialized macrophages (epithelioid cells) by 23%, an increase in histiocytes and fibroblasts of more than triple, increase in the number of small lymphocytes population of more than double, and a decrease of plasma immunocompetent cells number by double (Table 4). Such dynamics characterized the processes of the gradual silencing and delimitation of inflammation after 4 months of the experiment. In general, we can conclude for the group that the model of bone tuberculosis was well worked out, and the development of specific inflammation of the metaepiphyseal area of the distal femur with a subtotal lesion was achieved.

#### 3.5.2. Group 2: Autoplastic Bone Surgery

**Figure 6:**
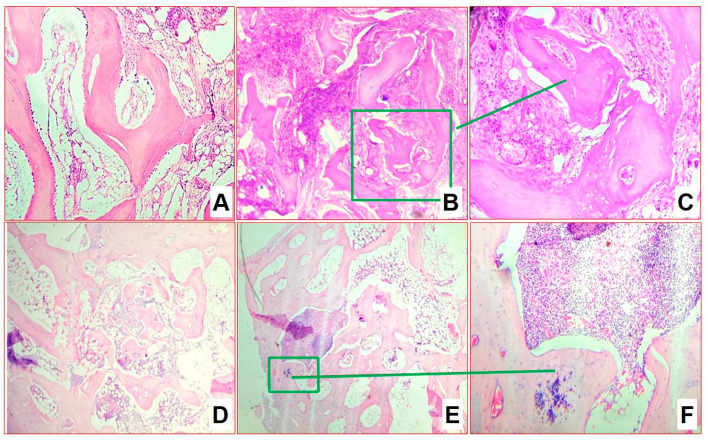
Morphological changes in group 2 after 2 (**A**–**C**) and 4 (**D**–**F**) months of infection (H&E staining): (**A**) osteoblasts surrounding autologous bone with phenomena of involved osteogenesis in places of mesenchyme growth (×200); (**B**,**C**) in areas of inflammation, phenomena of autologous bone sequestration (×100 (**B**) and ×200 (**C**)).

Two months after infection in the lateral and central part of the epiphysis zone, a few areas of lymphoplasmacytic infiltration and predominantly macrophage granulomas with rare multinucleated cells were visualized. The activity of specific inflammation was high, but the total affected area did not exceed 40% of the epiphysis. Specific inflammation was predominantly located along the lateral walls of the induced defect, around the autograft structures. The autograft itself retained the skeleton of the beams, some of them are actively outgrowing with new replacement bone tissue. In addition, one can observe growth islands of mesenchyme with osteogenesis, the activity of osteoblasts, and the sprouting of immature and mature granulation tissue, as well as filling the inter-bar spaces with hypoplastic bone marrow (Figure 6A). In the areas of inflammation, regions of autologous bone sequestration were retained (Figure 6B,C).

After four months, there is a complete reduction in specific inflammation and the epiphysis area is remodeled. In particular, autologous bone is fully involved in the creation of a new trabecular structure of the epiphysis (Figure 6D), in the interbody bone marrow with an uneven distribution of zones of hypo- and hypercellularity (Figure 6E,F). Small sequesters with the phenomena of resorption, minor areas of inflammation around the autologous bone are found in places.

**Figure 7:**
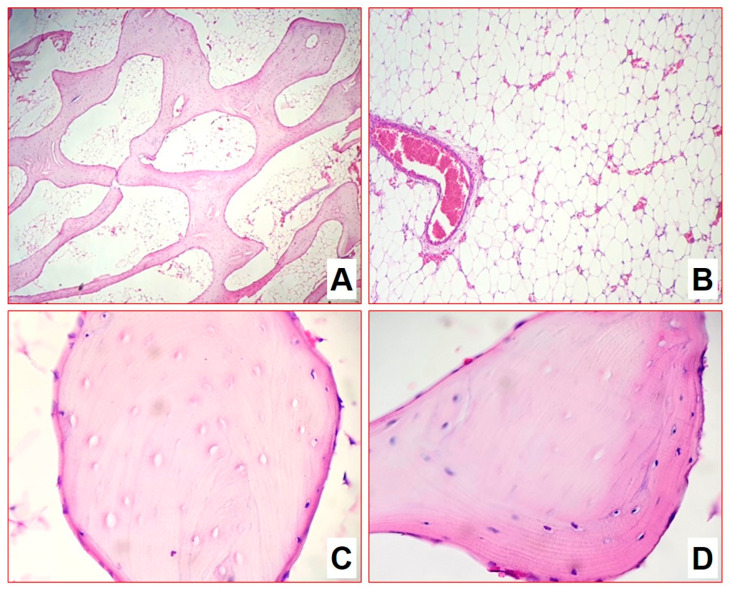
Morphological changes in group 2 after 6 months of infection (H&E staining): (**A**) bone beams in epiphysis zone (×100); (**B**) blood vessels in epiphysis zone (×100); (**C**,**D**) bone beam with empty osteocyte lacunae (autograft remnants) (**C**); and this with lamellar overgrowth of osteocytes (**D**) (×200).

After six months, the architectonics of the epiphysis–metaepiphysis area were restored; the bone network was represented by mature bone beams of normal thickness. The bone beams formed a looped network uniformly filling the epiphyseal area, while the autograft structures were not determined (Figure 7A). At this stage, an almost-complete reduction in specific inflammation was indicated. No manifestations of inflammatory processes and areas of fibrosis were observed. Bone marrow has a normal histological structure and is aplastic in the epiphysis and metaepiphysis area. Blood vessels were normal in number and full of blood (Figure 7B). In the cell areas of the diaphysis in the bone marrow, the cells of all hematopoietic growths at all stages of development were determined. In the diaphysis, bone beams with empty osteocyte lacunae were visualized, around which new bone tissue with living osteocytes, with a small number of osteoblasts on the surface (autograft remnants) built up (Figure 7C,D). The ratio of hematopoietic tissue to adipocytes was 33/67%. The average number of vessels in the field of view in the area of surgical intervention and in the area of the epiphysis contralateral (×1000) was 1.2 and 0.7, respectively.

#### 3.5.3. Group 3: Plastic Surgery with Composite Scaffold

**Figure 8:**
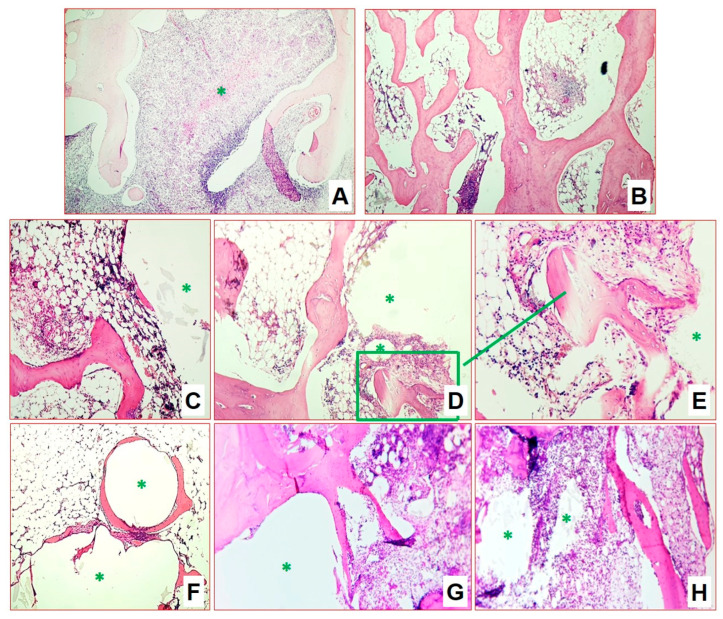
Morphological changes in group 3 after 2 months of infection (H&E staining, ×100): (**A**) macrophage–lymphocytic infiltrates with caseous necrosis (*), among deformed bone beams; (**B**) single submilliar productive macrophage–lymphocytic granulomas among deformed bone beams; (**C**,**D**) marked areas of implant material (*) surrounded by newly formed mature and growing bone beams, also sprouting into the pore structure, in the surrounding tissue hypoplastic bone marrow; and (**E**–**H**) perifocal areas around implant elements (*) with inflammation and remodulation of bone beams.

As in group 2 (AP), after 2 months, the epiphysis zone showed preservation of the bone structure architectonics. In the lateral and central part, a few areas of lymphoplasmacytic infiltration and predominantly macrophage granulomas with rare multinucleated cells, and foci with decay were visualized. The activity of specific inflammation, as for group 2 (AP), was high, but the total affected area also did not exceed 40% of the epiphysis (Figure 8A,B). As in the case of AP, specific inflammation was predominantly located along the lateral walls of the induced cavity, around the implant structures. The implant itself retained its skeleton both in terms of size and pore structures. On the outside, one can observe the formation of a thin connective tissue capsule. No rejection, inflammation or resorption reactions were observed during this time. The material demonstrated high inertness relative to the body tissues. Active sprouting of the pores by the newly formed bone tissue was detected. In addition, growing islands of mesenchyme with osteogenesis, and high activity of osteoblasts, as well as sprouting of immature and mature granulation tissue in the interbeam spaces, and filling with hypo- and hyperplastic bone marrow, were observed (Figure 8C,D). In the perifocal areas around the implant, there was an active process of bone structure remodeling and formation of primary bone callus after the bone necrectomy (Figure 8E–H).

**Figure 9:**
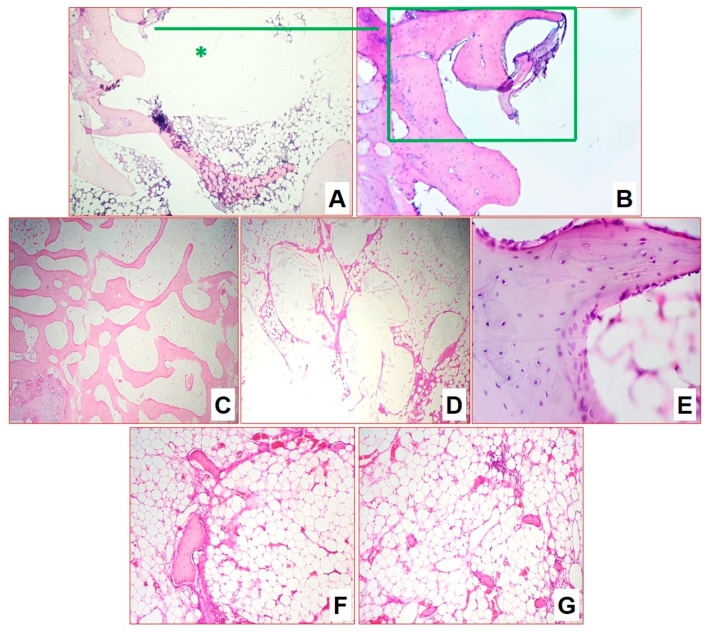
Morphological changes in group 3 after 4 (**A**,**B**) and 6 (**C**–**G**) months of infection (H&E staining): (**A**,**B**) implant elements grew into mature bone beams ((**A**), ×100), sprouting through material pores ((**B**), ×200); (**C**) bone beams in epiphysis zone (×100); (**D**) implant remnants in epiphysis zone (×100); (**E**) bone beam next to the implant with active osteoblasts on the surface (×200); (**F**) stromal bands in the metaepiphysis zone (×100); and (**G**) full-blooded vessels and newly formed beams (×100).

After four months, the specific inflammation was residual and persisted as single small infiltrates and granulomas without necrotization. Inflammatory activity was minimal, occupying not more than 5% of the area. The implantation zone was represented by mature bone beams growing and sprouting into part of the implant; a slight rim of connective and mesenchymal tissue with active osteoblasts was retained along the edges (Figure 9A,B). In addition, in certain places the growing beams, osteoclasts, participating in the processes of remodeling in the area of the formed bone callus were visible. The inter-beam spaces were filled with bone marrow with a significant number of hypercellular zones.

At six months after infection, there was a complete reduction in specific inflammation. Inflammatory infiltration in the specimens was weak and nonspecific, associated with the formation of a capsule around the implanted material. The cortical bone in the epiphysis zone was represented by mature lamellar bone tissue with well-visualized osteonic structures. The metaepiphysis plate was well expressed and represented by mature bone beams. Bone marrow in the area of the epiphysis, metaepiphysis and most of the diaphysis was aplastic, without manifestations of inflammatory processes and areas of fibrosis. In the cellular areas of the diaphysis in the bone marrow, cells of all hematopoietic growths at all stages of development were determined. Histoarchitectonics of the epiphysis zone were close to the normal structure. There were beams in the epiphysis of normal histological structure, forming a looping network; on single beams there were areas with active osteoblasts on the surface (Figure 9C).

The inter-bar space of the epiphysis zone was represented by reticular stroma with adipocytes; there were single bone marrow cells, which were mostly represented by lymphocytes and granulocytes, and rarely monocytes/macrophages. There were local areas with increased cellularity. The vascular network was developed, represented by vessels of different calibers of venous and arterial types. The number of vessels was increased, and they were full-blooded. The average number of vessels in the field of view per group in the area of surgical intervention and in the area of epiphysis contralateral (calculated at ×1000) was 1.56/0.96.

The implantation zone was represented by rounded cavities with lamellar or loose implant remnants surrounded by a thin connective tissue capsule of 2–5 layers of cells (Figure 9D). Bone marrow stromal tissue had sprouted between the pores of the implanted material, in some places represented by a connective tissue capsule, and in some places with a predominance of adipocytes and stromal cells, and the newly formed bone beams had also sprouted between the pores. Around the thin connective tissue capsule surrounding the implant, there were newly formed beams repeating the contour of the implant (Figure 9D). On single newly formed beams, there were areas of active osteoblasts on the endosteal surface that testified to the prolonged activation of osteogenesis (Figure 9E). The number of thin-walled, full-blooded vessels was increased in this area (Figure 9F,G); however, active mesenchyme was not defined any more.

In general, a positive result was achieved not only in the treatment of tuberculosis, but also in the remodulation of the bone structures of the epiphysis zone.

### 3.6. Quantitative Assessment of Bone Regeneration

**Figure 10:**
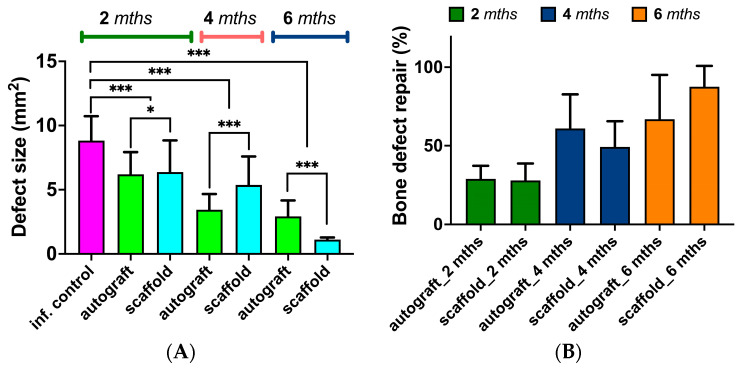
In vivo bone repair assessment with application of micro-CT images. (**A**) Defect size change during the time of in vivo experiment. (**B**) Bone repair at the site of defect (necrectomy). For assessment of bone defect repair, the size of defect at each time point was referred to the defect size of infected control. The values are presented as mean ± SD (*n* = 16). Statistical analysis: * *p* ≤ 0.05; *** *p* ≤ 0.001.

The regeneration of bone tissue after necrectomy followed by autograft or PCL/PGlu-NCC + rMSCs scaffold application was quantitatively assessed with application of data obtained by micro-CT and histology. In both cases, images of bone slices in the defect site were analyzed to evaluate the size of the defect and bone tissue repair. One can observe (Figure 10A) the decrease in defect size during in vivo experiment. In the fourth month after implantation, the size of the defect was less in the case of autograft application. At the same time, the PCL/PGlu-NCC + rMSCs scaffold showed a minimal area of post-necretomy cavity after six months of the experiment.

Bone repair in the site of the necrectomy-caused defect was assessed by referring the size of the defect at each time point to that of the infected control (without any plastic surgery). It was observed that the bone tissue repair at the site of defect was ongoing during all experiments (Figure 10B) and showed its maximum value in the case of the PCL/PGlu-NCC + rMSCs scaffold application after six months of the experiment.

**Figure 11:**
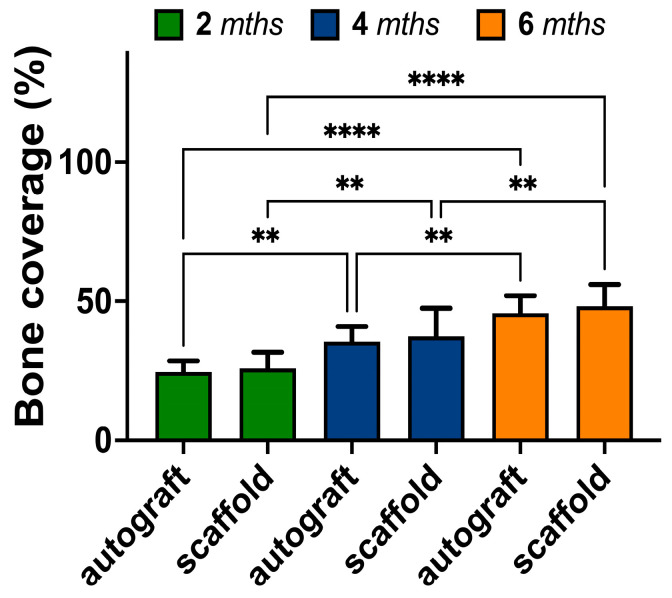
Bone coverage at the site of autografting/implantation evaluated from corresponding histological images. The values are presented as mean ± SD (*n* = 10). Statistical analysis: ** *p* ≤ 0.01; **** *p* ≤ 0.0001. The difference between autograft and scaffold at each time point was statistically non-significant.

In addition to the micro-CT measurements, bone regeneration in the site of defect was also evaluated from histological images. It was observed (Figure 11) that the bone coverage in the cases of both autografting and implantation was increasing. The area of bone tissue detected after 6 months was significantly greater than that after 2 months. However, according to calculations based on histological data, the bone formation was similar when the autograft or scaffold were applied.

## 4. Discussion

Bone tuberculosis is one of the extrapulmonary forms of tuberculosis. Traditional treatment for bone tuberculosis includes necrectomy combined with anti-TB chemotherapy [10]. Currently, bone autografts are the gold standards in the repair of bone defects, including the treatment of bone tuberculosis [58]. However, due to limitations in availability and the pain associated with autograft harvesting, various biodegradable implants have been actively developed [59]. The latter can fill the defect and provide the necessary mechanical function at the initial stage, and then be replaced by own bone tissue as the implant degrades. In our previous studies [43,50] we developed 3D-printed composite scaffolds composed of PCL as the dispersion phase and NCC modified by PGlu as the dispersed phase. These scaffolds were optimized towards their physicochemical characteristics and showed inspiring biological properties during in vivo experiments with healthy rabbits [43]. It was shown that bone tissue regeneration of such scaffolds was most in the case of their pre-seeding with MSCs. In the presented research, we investigated the potential of developed 3D-printed PCL/PGlu-NCC + rMSCs materials as scaffolds for bone regeneration after the necrectomy of bone tuberculosis. For the evaluation of materials under study as scaffolds for bone repair, they were compared with bone autografts.

The model of bone tuberculosis was successfully generated in rabbits involved in the experiment. This was proved by an immunological subcutaneous test, the real-time PCR of the bone tissue samples extracted during necrectomy, and histomorphological analysis of the bone specimens from animals in control group. In addition, biochemical study confirmed the presence of ceruloplasmin and elastase, which are known markers of the acute inflammatory process. Elastase could also serve as a regulator of the inflammatory response and can act both as a pro-inflammatory and anti-inflammatory agent [60].

Biochemical analysis of blood taken from experimental animals is known to be a useful tool for the evaluation the inflammation and bone regeneration processes [61,62]. Two months after infection in groups 2 (with autografting) and 3 (with composite scaffold surgery), the decrease in ceruloplasmin levels and an increase in the activity of purine metabolism enzymes (total ADA and ADA-1), regulating the level of adenosine, were detected. Locally released adenosine mediates its physiological and pharmacological actions through interactions with G-protein receptors, which are known to be involved in the regulation of osteoclast differentiation and function as well as in osteoblast differentiation and bone formation [63]. All four adenosine receptors (A_1_, A_2A_, A_2B_ and A_3_) are expressed by bone marrow stromal cells and differentiated osteoblasts [64,65]. In group 2, the revealed correlation between the activity of ADA-2 and elastase indirectly illustrates the ability of adenosine to inhibit elastase secretion by binding to the A_2A_ receptor, which induces cyclic adenosine monophosphate (cAMP) formation [66]. Correlations between the molecules involved in the regulation of cAMP-mediated metabolism were revealed in group 3. Albumin selectively and reversibly inhibits the TNF-induced fall in the level of cAMP [67], and EL secretion is controlled by adenosine [68].

Four months after infection in group 2, along with preservation of high activity of total ADA and ADA-1, the concentration of nonspecific alkaline phosphatase (ALPL) was also found to increase. Increased alkaline phosphatase activity could serve as an indicator of changes in bone structure. This is likely to be true, because our earlier experiments with healthy rabbits showed that such changes in alkaline phosphatase activity are not associated with possible liver diseases. Increased expression of this enzyme by osteoblasts is the reason for the elevation of its level in the blood. Therefore, the determination of the concentration of alkaline phosphatase is an informative marker of bone remodeling [69]. In contrast, only high ADA-1 activity was detected in group 3. Also, in this group, the correlations in the regulation of cAMP-mediated metabolism were preserved. The negative correlations between the activity of total ADA, ADA-1 and alkaline phosphatase concentration indirectly shows the possibility of alkaline phosphatase inhibition through the growing concentration of adenosine [70].

Six months after infection in group 2, a high concentration of non-specific alkaline phosphatase (ALPL) and high activity of total ADA and ADA-1 were preserved. In group 3, an increase in ADA-2 activity without an increase in total ADA and ADA-1 activity, as well as a balanced regulation of cAMP-mediated metabolism, was revealed. Also in this group, a negative correlation between ADA-2 activity and RANKL concentration was detected. An increase in ecto-ADA-2 activity is associated with a decrease in extracellular adenosine levels and the adenosine-associated activation of the A_2A_ receptor. This process was previously shown to counteract osteoclastogenesis reducing bone resorption in a collagen-induced mouse model of rheumatoid arthritis [65,71]. The binding of both transmembrane and soluble forms of RANKL to RANK leads to the trimerization of the receptor, which through a complex chain of adaptor molecules activates various signaling pathways going to nuclear factor kappa B (NF-κB). In turn, this leads to the initiation of osteoclastogenesis from precursor cells and the activation of mature osteoclasts [72], while the A_2A_ receptor mediates the activation of NF-κB [73].

Overall, the concentration of biochemical markers revealed the positive dynamics of the bone-formation process as well as absence of negative effects on normal metabolism within experimental animals.

Another tool to investigate the changes in bone tissue of experimental animals was histomorphological analysis. This method also clearly showed that the model of tuberculous inflammation was successfully realized in all rabbits under study. In the control group, inflammation reached its maximum at two months after infection and was characterized by the formation of extensive necrosis zones with loose macrophage–lymphocytic–plasmacytic infiltrates on the periphery of necrotized zones with an abundance of *M. tuberculosis*. Inflammation affects 80% of the epiphysis. However, the beam framework, except minor zones of sequestration, was preserved. After 4 months of the experiment, a subsiding of inflammation, and the isolation of necrosis foci, with the formation of a dense macrophage–histiocytic and/or macrophage–lymphocytic shaft on the periphery of the decay foci, were detected within histological sections of bones.

In the groups with autografting (group 2) and the scaffold implantation (group 3), a minor active inflammation was detected within two months after infection and subsequent anti-tuberculosis chemotherapy. It localized mainly on the periphery of the surgical defect and around the autograft or implant. Within four months after infection, the tuberculous inflammation was practically reduced. The formation of bone calluses was observed in both cases of autograft or composite implant application. In the case of the composite scaffold implantation (group 3), the number of existing and forming sequesters was significantly lower even at 4 months after infection. This observation reflected the absence of inflammation in the area implantation. Also, after 4 months of the experiment, the more pronounced formation of red bone marrow at the site of implantation in group 3 (scaffold implantation) was detected compared to group 2 (autografting). It was notable that after six months post-infection, groups 2 and 3 showed the complete reduction in specific and nonspecific inflammation. The tissue structure within the epiphyseal–metaepiphyseal region was restored. The bone beam network was represented by mature bone beams of normal thickness. In the case when PCL/PGlu-NCC-rMSCs scaffolds were used for plastic surgery, the implant framework preservation was visualized. There were newly formed bone beams as well as bone marrow stromal tissue sprouts between the pores of the implanted material. A graft-rejection reaction and active resorption of the material were not observed. This shows the absence of toxicity of the applied materials. The absence of the PCL/PGlu-NCC + rMSCs composite material’s toxicity could be explained by the non-toxicity of the components of this scaffold. PCL was approved by the FDA for various medical applications, including implantation [74], and was investigated as long-termed drug-releasing implant [75]. Cellulose in different forms and PGlu were also investigated and approved as components of materials for in vivo medical application [76,77]. Importantly, the presence of active osteoblasts on the surface of the newly formed beams indicated the active osteogenesis process.

Both discussed histomorphological data and micro-CT images of the rabbit bone specimens derived at 2, 4 and 6 months after infection with a multi-drug-resistant strain of *M. tuberculosis* indicated successful bone regeneration using both an autograft and a composite PCL/PGlu-NCC-rMSCs scaffold. The substitution of the cavity with newly formed bone tissue was shown by micro-CT. According to earlier published studies, the scaffolds based on aliphatic polyesters with osteoconductive properties such as PLA/BMP-2 scaffolds or PLA/HA/collagen-MSCs are known to provide bone coverage of 30–34% after one month of implantation [78,79]. Also, it was shown that PLGA/β-TCP composite scaffolds were completely degraded and substituted with new bone within 12 weeks of an experiment [47]. This is much faster than was observed in our work. However, it should be noted that non-infected animals were applied in the above-mentioned studies. Thus, one can expect the difference in regeneration rate due to inflammation process.

The results of our qualitative micro-CT based assessment of bone tissue repair showed that more than 80% of the bone defect caused by tuberculosis necrectomy was regenerated during six months of the experiment in the case of PCL/PGlu-NCC-rMSC scaffold application. It was even more than in the case of the autograft. However, considering the high values of standard deviation in such experiments, we can conclude that the autograft and scaffold gave nearly the same results. The analysis of histological images allowed us to evaluate bone coverage at the defect site. Despite the observation that new bone tissue structures appeared during all periods of the in vivo experiment, there was no difference between scaffold and autograft. In the current study, the bone coverage observed after 6 months of the in vivo experiment was about 50%. This value is close to the previously published results [80]. In the cited study, bone coverage was about 55% after 6 months post-implantation of the β-TCP-based scaffold coated with autologous bone marrow into the femoral bones of healthy rabbits. Our previous examination of the PCL/PGlu-NCC-rMSCs composite scaffold in healthy rabbits revealed about 33 and 55% of bone coverage after one and three months of implantation [43]. Thus, we can conclude that bone regeneration in the case of rabbits, which were affected by tuberculosis, was less intensive than that in the case of healthy animals. Nevertheless, the closeness of bone coverage values found by us and the researchers who studied healthy rabbits for the same period [80] indicates the success of the bone healing process. Thus, we have showed the possibility of developed scaffolds application for the regeneration of bones after necrotomy of infected tissue.

Scaffold degradation rate is an important factor, which affects the effectiveness of the bone-healing process. Despite PLGA, PLA and PCL all being biodegradable polymers, PCL degradation rate is slowest in the list [81]. It is known that PCL degrades due to hydrolytic surface erosion in aqueous environments [82]. Moreover, the polymer hydrolysis in vivo may be affected by different kinds of esterases [83]. Depending on the molecular weight and implant volume, PCL scaffolds can retain their shape and provide sufficient mechanical support from several months to several years [81,84]. For instance, Sun et al. showed that PCL monolithic cylindrical implants of 2.3 mm in diameter and 2.3 cm in length kept their shape in vivo within two years [81]. According to histomorphological analysis in this study, the scaffold framework was also not degraded totally in the six-month experiment. However, according to the literature, the PCL composites filled with micro- or nanocrystalline cellulose demonstrate an increased degradation rate of the matrix [85,86]. The accelerated degradation in this case is explained by two simultaneous processes. First, the release of the hydrophilic filler from hydrophobic matrix while its degradation takes place. Second, the formation of voids associated with filler release increases specific surface area due to the increase in porosity of scaffold walls. The latter improves water and enzyme diffusion inside the scaffold. In turn, the better wettability of PCL composites containing micro- and nanocrystalline cellulose enhances adsorption of the enzymes that has a great impact on the enzyme-mediated degradation. As it was mentioned at the discussion of the histological analysis results, the total degradation of the PCL/PGlu-NCC scaffold was not reached in this study. However, it was expected to be faster compared to more hydrophobic initial PCL.

Compared to studies of the relatively fast-degrading PLA- and PLGA-based materials for the substitution of bone defects after tuberculosis removal [47,78], PCL-based materials are perspective for providing long-term scaffold stability. At the same time, NCC modified by PGlu was shown to be responsible for its osteogenic effect [50]. Thus, PCL/PGlu-NCC scaffolds could be considered as materials for the regeneration of large bone defects, when long-term scaffold stability is needed to provide mechanical support for surrounding tissue and cells ingrowth.

Overall, biochemical, histological and micro-CT data in our study indicated that both autograft plastic surgery and composite scaffold implantation gave positive results towards the remodulation of bone structures of the epiphyseal zone. A decrease in the activity of tuberculous inflammation in the bone was achieved by effective chemotherapy, while autografts and scaffolds provide a high level of osteogenetic potency. Comparable results obtained in the discussed two different variants of surgery argue in favor of the application of the studied composite material in clinical practice, since in this case there is no need for additional surgical manipulations on the preparation of bone autograft. In addition, in the case of scaffold application, the time of surgical intervention is reduced and intraoperative blood loss decreases. Long-term stability of PCL together with osteinductive properties provided by PGlu-NCC allowed the formation of a new trabecular framework, while preserving the skeleton of the implant material. This favors the higher strength of the damaged bone and reduces the possible complications associated with the sequestration and non-adaptability of autologous bone. Therefore, the developed composite scaffolds can be a material of choice in cases when significant volumes of material are required, but the autograft application is complicated or impossible.

The results reported in current paper should be considered in the light of some limitations. One of such is that sample size in the infected control group could be increased to perform better statistical evaluation. Also, during the quantitative assessment of the bone growth with micro-CT, the size of the defect was referred to the infected control group of animals only after two months post-infection. At such calculations, it would be more precise to refer defect size at each time point to the infected control at the same time points. However, this could be an issue due to the bad survivability of infected animals. Another limitation to be considered is the compliance of the scaffold mechanical properties and the degradation rate to those of the corresponding type of bone. The scaffold used in this study was designed for the reconstruction of bones with compression moduli up to 100 MPa. It should be specially noted that we did not use local chemotherapy against tuberculosis in our study, but only a systemic one. However, in many studies the possibility of scaffold application as a local antibiotic delivery system was successfully shown [12,47]. Therefore, our further plans include the loading of presented PCL-based composite scaffolds with anti-tuberculosis drugs to enhance the efficacy of treatment. In addition, future studies on the implantation of larger-sized scaffolds, including the possibility of using them as spacers between two separated bone fragments, are needed to determine the dimensional limitations in the use of the developed composite scaffolds.

## Figures and Tables

**Table 1:** Monitoring the body weight of animals during the experiment.

Group	Average Rabbit Weight (g)				
	Initial Weight	18 Days	2 Months	4 Months	6 Months
1 (IC)	3518 [3256; 3676]	3348 [3164; 3572]	2949 (*p* = 0.02 *) [2816; 3082]	2713 (*p* = 0.04 **) [2713; 2713]	–
2 (AP)			3809 (*p* = 0.02 *; *p* = 0.003 **) [3486; 4025]	4251 (*p* = 0.00003 *; *p* = 0.00001 **) [3943; 4435]	3995 (*p* = 0.03 *; *p* = 0.007 **) [3635; 4395]
3 (SI)			3912 (*p* = 0.0008 *; *p* = 0.0001 **) [3574; 4144]	3959, (*p* = 0.002 *; *p* = 0.0001 **) [3646; 4356]	4270 (*p* = 0.01 *; *p* = 0.001 **) [3660; 4280]

*—differences are significant compared to initial data; **—differences are significant compared to 18 days after infection.

**Table 2:** Monitoring of biochemical markers of inflammatory response and markers of bone tissue metabolism.

Biochemical Parameter	Initial Values	Groups										
		1 (*n* = 4)			2 (*n* = 12)				3 (*n* = 15)			
		Time Periods after Infection for Biochemical Screening										
		18 Days	2 Months	4 Months	18 Days	2 Months	4 Months	6 Months	18 Days	2 Months	4 Months	6 Months
EL (IU)	293.4 [251.9; 323.3]	347.7 (*p* = 0.0008 *) [293.4; 423.8]	239.0 [239.0; 239.0]	130.0 [130.0; 130.0]	347.7 (*p* = 0.0008 *) [293.4; 423.8]	297.0 [233.9; 459.2]	298.6 [279.8; 364.0]	293.3 [249.9; 396.5]	347.7 (*p* = 0.0008 *) [293.4; 423.8]	484.6, (*p* = 0.000001 *; *p* = 0.01 **; *p* = 0.01 **^#^**; *p* = 0.02 **^&^**) [384,6; 532,5]	342.2 [288.0; 434.7]	362.2 (*p* = 0.056 *) [282.5; 402.0]
TP (g/L)	71.0 [66.0; 77.0]	65.6 (*p* = 0.05 *) [62.5; 70.4]	64.5 [63,0; 66,0]	67.0 [67.0; 67.0]	65.6 (*p* = 0.05 *) [62.5; 70.4]	66.0 [64.0; 71.0]	77.0 (*p* = 0.015 *; *p* = 0.002 **) [72.5; 86.5]	76.0 (*p* = 0.05 **) [71.0; 82.5]	65.6 (*p* = 0.05 *) [62.5; 70.4]	65.0 [63.0; 72.0]	72.0 (*p* = 0.05 **) [67.0; 82.0]	70.0 [66.0; 71.0]
AL (g/L)	47.0 [45.0; 50.0]	46.0 [45.0; 49.0]	46.0 [46.0; 46.0]	42.0 [42.0; 42.0]	46.0 [45.0; 49.0]	46.0 [44.0; 49.0]	50.5 (*p* = 0.01 *; *p* = 0.0002 **) [49.0; 51.0]	47.0 [46.0;49.5]	46.0 [45.0; 49.0]	47.0 [45.0; 50.0]	48.0 (*p* = 0.01 **) [47.0; 52.0]	47.0 [46.0; 47.0]
CP (g/L)	0.34 [0.28; 0.45]	0.42 (*p* = 0.01 *) [0.35; 0.52]	0.46 [0.45; 0.48]	0.95 [0.95; 0.95]	0.42 (*p* = 0.01 *) [0.35; 0.52]	0.32, (*p* = 0.0009 *; *p* = 0.03 **) [0.26; 0.39]	0.23 (*p* = 0.00001 **) [0.15; 0.24]	0.28 (*p* = 0.01 **) [0.25; 0.34]	0.42 (*p* = 0.01 *) [0.35; 0.52]	0.33 (*p* = 0.0007 **; *p* = 0.01 **^#^**) [0.24; 0.38]	0.26 (*p* = 0.008 *; *p* = 0.000008 **; *p* = 0.03 **^&^**) [0.24; 0.28]	0.35 [0.29; 0.39]
Total ADA (U/L)	9.6 [6.8;11.6]	9.8 [6.7; 12.2]	17.0 [10.2; 23.9]	27.7 [27.7; 27.7]	9.8 [6.7; 12.2]	14.9 (*p* = 0.04 *) [6.9; 18.4]	17.0 (*p* = 0.001 *; *p* = 0.006 **) [11.3; 22.9]	13.8 [8.1; 17.7]	9.8 [6.7; 12.2]	13.4 (*p* = 0.0027 *; *p* = 0.02 **) [8.4; 16.2]	11.6 [8.8; 17.0]	11.6 [5.4; 14.0]
ekto-ADA-1 (U/L)	8.5 [6.6; 9.4]	8.3 [5.1; 11.2]	13.8 [7.7; 19.9]	23.5 [23.5; 23.5]	8.3 [5.1; 11.2]	12.0 (*p* = 0.004 *) [5.5; 17.0]	15.4 (*p* = 0.008 **) [8.8; 18.4]	10.5 [6.7; 15.8]	8.3 [5.1; 11.2]	12.1 (*p* = 0.004 *; *p* = 0.02 **) [7.9; 14.3]	10.6 (*p* = 0.02 *) [9.1; 17.0]	6.0 [3.1; 11.4]
ekto-ADA-2 (U/L)	0.45 [0; 2.0]	0.8 [0; 1.4]	3.3 [2.5; 4.0]	4.1 [4.1; 4.1]	0.8 [0; 1.4]	0.55 [0; 2.7]	0 [0; 3.6]	1.3 [0; 3.2]	0.8 [0; 1.4]	0.75 [0; 1.9]	0.35 [0; 1.8]	3.3 (*p* = 0.004 *; *p* = 0.004 **) [2.6; 3.5]
RANKL (ng/L)	99.7 *n* = 12 [87.5; 102.8]	76.7 (*p* = 0.002 *) *n* = 14 [57.6; 91.4]	101.0 (*p* = 0.04 *; *p* = 0.04 **) *n* = 2 [99.0; 103.0]	83.2 *n* = 2 [83.2; 83.2]	76.7 (*p* = 0.002 *) *n* = 14 [57.6; 91.4]	83.7 *n* = 12 [70.3; 94.3]	86.3 *n* = 8 [82.5; 92.4]	89.4 *n* = 4 [72.1; 92.4]	76.7 (*p* = 0.002 *) *n* = 14 [57.6; 91.4]	83.5 (*p* = 0.02 *) *n* = 15 [64.4; 92.5]	84.5 *n* = 10 (*p* = 0.04 **) [66.5; 100,0]	103.0 *n* = 5 (*p* = 0.01 **) [95.5; 106.0]
ALPL (ng/L)	0.32 *n* = 11 [0.2; 1.1]	0.13 *n* = 5 [0.1; 0.23]	*n* = 0	0.22 *n* = 2 [0.22; 0.22]	0.13 *n* = 5 [0.1; 0.23]	0.32 *n* = 5 [0.28; 0.62]	1.12 (*p* = 0.026 *; *p* = 0.04 **) *n* = 8 [0.54; 1.47]	1.35 (*p* = 0.04 *) *n* = 4 [0.6; 2.13]	0.13 *n* = 5 [0.1; 0.23]	0.24 *n* = 8 [0.18; 0.33]	0.42 *n* = 9 (*p* = 0.046 **^&^**) [0.35; 0.65]	0.64 *n* = 4 [0.36; 1.87]

Data are given as median and interquartile range. Abbreviations: EL—elastase, TP—total protein, AL—albumin, CP—ceruloplasmin, total ADA—total adenosine deaminase, ectoADA-1—ecto-adenosine deaminase-1, ectoADA-2—ecto-adenosine deaminase-2, RANKL—nuclear factor receptor activator kappa B ligand, ALPL—non-specific alkaline phosphatase. *—differences are significant compared to initial values; **—differences are significant compared to 18 days after infection, ^#^—differences are significant compared to Group 1 (infection control), ^&^—differences are significant compared to Group 2 (autoplastic surgery).

**Table 3:** Qualitative analysis of micro-CT images for several indicators.

Group *	Indicator (2/4/6 Months, Respectively) 1: The Indicator Is Positive; 0: The Indicator Is Negative					
	Bone Trabeculae Sprouting	Structurality of Bone Beams	Bone Callus	Marginal Bone Growths	Resorption Zone	Sclerosis Area along the Defect
1 (IC)	0/1	0/0	0/1	1/1	1/1	0/0
2 (AP)	1/1/1	0/1/1	1/1/1	1/1/1	1/1/1	0/1/0
3 (SI)	1/1/1	0/1/1	1/1/1	1/1/0	1/1/0	1/0/0

*—IC—infection control; AP—autoplastic; SI—scaffold implantation.

**Table 4:** Distribution of cells in the surrounding necrosis area for infection control group (calculated per 1000 cells).

Time, Months	Cells (%)					
	Macrophages	Fibroblasts	Lymphocytes	Plasmacytes	Eosinophiles	Multinucleated Cells
2	50.0	4.0	12.2	21.1	12.6	0.1
4	33.7	13.6	29.3	11.1	12.3	0

## Data Availability

Data are available within the article.

## Supplementary Materials

The following supporting information can be downloaded at: https://www.mdpi.com/article/10.3390/biomedicines11082229/s1, Figure S1: Micro-CT images for bone specimens extracted after 2 (A), 4 (B) and 6 (C) months of tuberculosis infection.
