# Supplementary material for: Comparison of Autografts and Biodegradable 3D-Printed Composite Scaffolds with Osteoconductive Properties for Tissue Regeneration in Bone Tuberculosis
Source: Biomedicines. 2023 Aug 8;11(8):2229. doi: 10.3390/biomedicines11082229 (PMC10452435; doi:10.3390/biomedicines11082229)
Supplement: Supplementary file 1 [file biomedicines-11-02229-s001.zip › biomedicines-2506285-supplementary.pdf]

Supplementary Materials

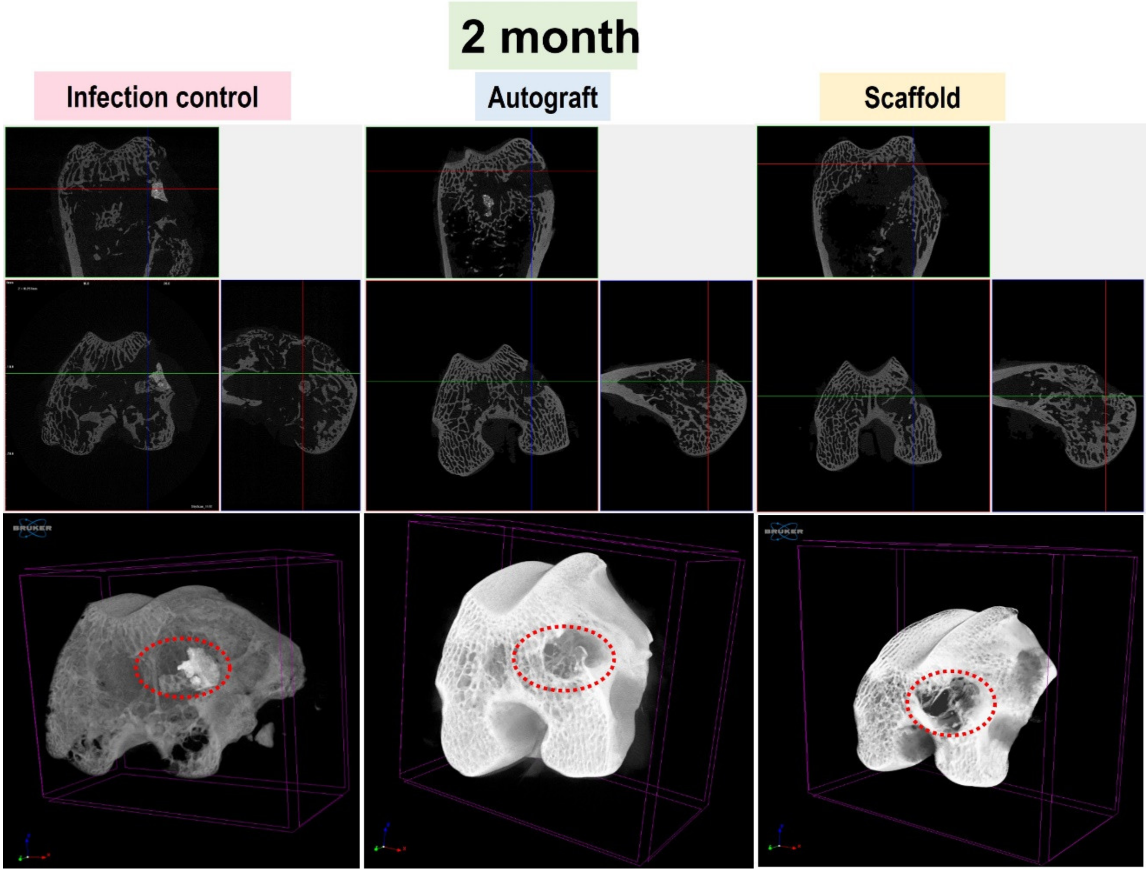

(**A**)

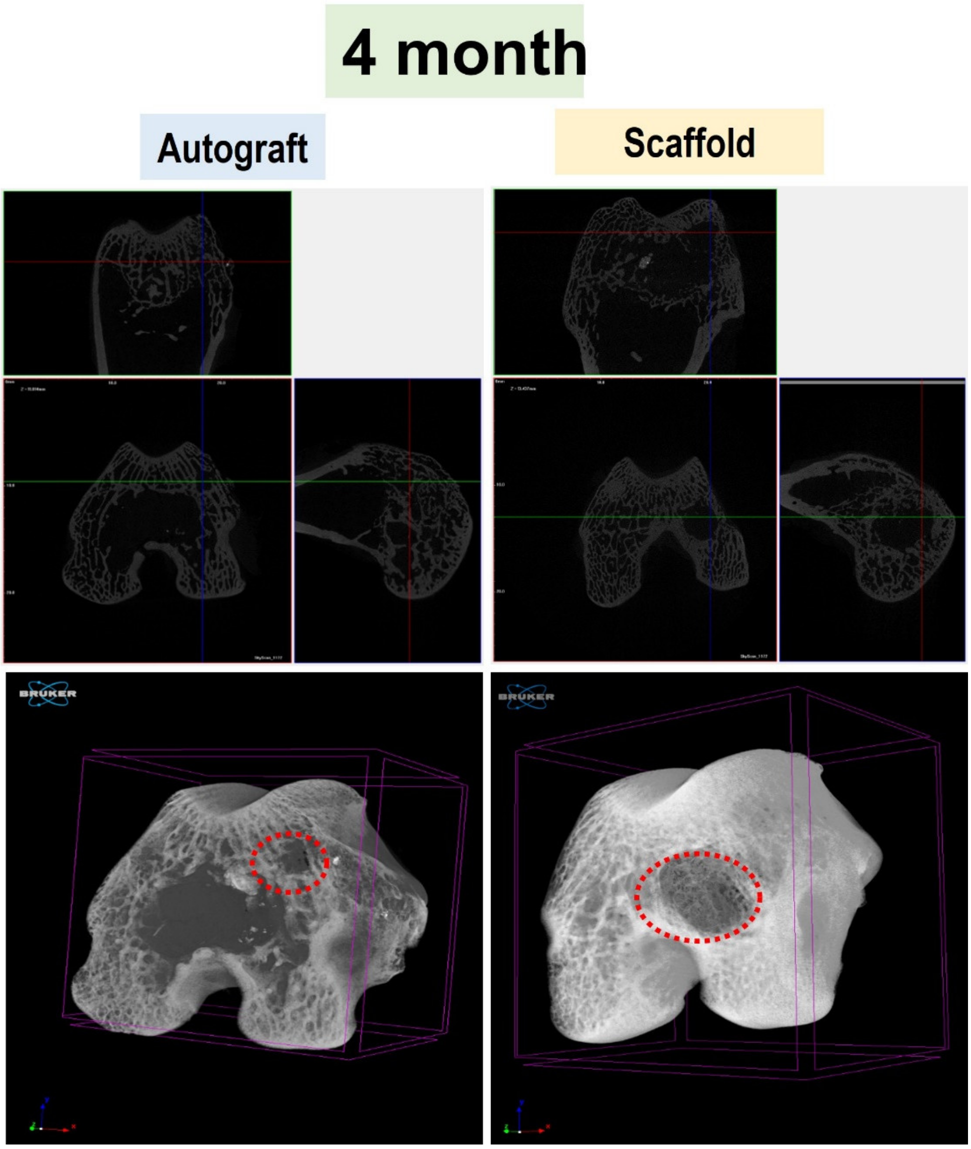

(**B**)

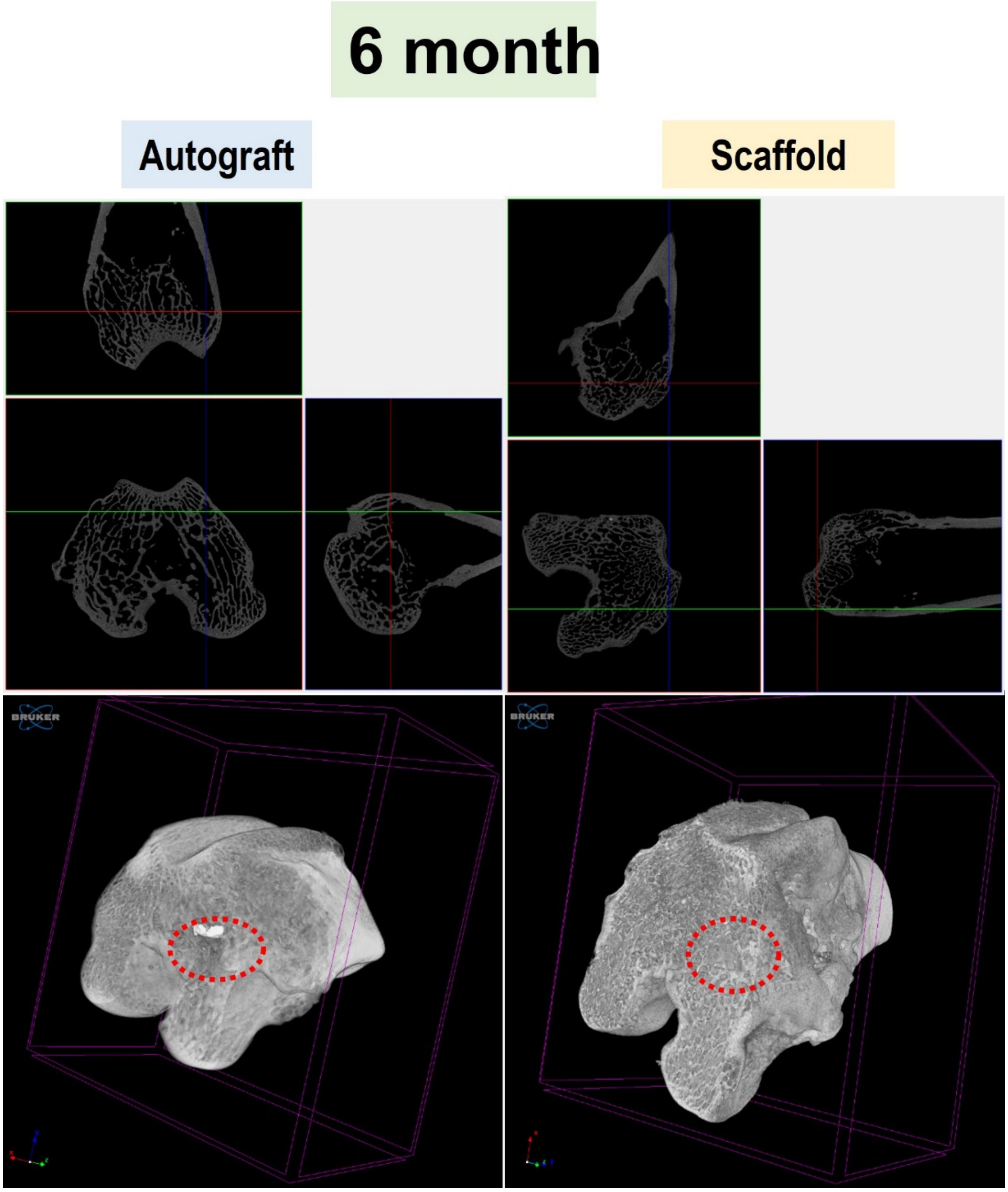

(**C**)

Figure S1. Micro-CT images for bone specimens extracted after 2 (**A**), 4 (**B**) and 6 (**C**) months of tuberculosis infection. Red circles show the zone of surgical intervention. Upper images were obtained from DICOM files with application of SkyScan DataViewer software, while bottom figures are 3D reconstructions prepared with SkyScan ctVOX software (ctVOX-v.3.2.0r1294, Brucker micro-CT, Billerica, MA, USA). Both programs are freely distributed software, suitable for Brucker micro-CT equipment.
